# Recalibration and Validation of the Charlson Comorbidity Index in Korean Incident Hemodialysis Patients

**DOI:** 10.1371/journal.pone.0127240

**Published:** 2015-05-18

**Authors:** Jae Yoon Park, Myoung-Hee Kim, Seung Seok Han, Hyunjeong Cho, Ho Kim, Dong-Ryeol Ryu, Hyunwook Kim, Hajeong Lee, Jung Pyo Lee, Chun-Soo Lim, Kyoung Hoon Kim, Kwon Wook Joo, Yon Su Kim, Dong Ki Kim, Clinical Research Center for End Stage Renal Disease (CRC for ESRD) Investigators

**Affiliations:** 1 Department of Internal Medicine, Seoul National University College of Medicine, Seoul, Korea; 2 Department of Dental Hygiene, College of Health Science, Eulji University, Gyeonggi-do, Korea; 3 Department of Epidemiology and Biostatistics, School of Public Health, Seoul National University, Seoul, Korea; 4 Department of Internal Medicine and Ewha Medical Research Institute, School of Medicine, Ewha Womans University, Seoul, Korea; 5 Department of Internal Medicine, Wonkwang University College of Medicine Sanbon Hospital, Gyeonggi-do, Korea; 6 Department of Internal Medicine, Seoul National University Boramae Medical Center, Seoul, Korea; 7 Department of Public Health, Graduate School, Korea University, Seoul, Korea; L' Istituto di Biomedicina ed Immunologia Molecolare, Consiglio Nazionale delle Ricerche, ITALY

## Abstract

**Background:**

Weights assigned to comorbidities to predict mortality may vary based on the type of index disease and advances in the management of comorbidities. We aimed to develop a modified Charlson comorbidity index (CCI) in incident hemodialysis patients (mCCI-IHD), thereby improving risk stratification for mortality.

**Methods:**

Data on 24,738 Koreans who received their first hemodialysis treatment between 2005 and 2008 were obtained from the Korean Health Insurance dataset. The mCCI-IHD score were calculated by summing up the weights which were assigned to individual comorbidities according to their relative prognostic significance determined by multivariate Cox proportional hazards model. The modified index was validated in an independent nationwide prospective cohort (n=1,100).

**Results:**

The Cox proportional hazards model revealed that all comorbidities in the CCI except ulcers significantly predicted mortality. Thus, the mCCI-IHD included 14 comorbidities with re-assigned severity weights. In the validation cohort, both the CCI and the mCCI-IHD were correlated with mortality. However, the mCCI-IHD showed modest but significant increases in *c* statistics compared with the CCI at 6 months and 1 year. The analyses using continuous net reclassification improvement revealed that the mCCI-IHD improved net mortality risk reclassification by 24.6% (95% CI, 2.5-46.7; *P*=0.03), 26.2% (95% CI, 1.0-51.4; *P*=0.04) and 42.8% (95% CI, 4.9-80.8; *P*=0.03) with respect to the CCI at 6 months and 1 and 2 years, respectively.

**Conclusions:**

The mCCI-IHD facilitates better risk stratification for mortality in incident hemodialysis patients compared with the CCI, suggesting that it may be a preferred index for use in clinical practice and the statistical analysis of epidemiological studies.

## Introduction

Although the prognosis of dialysis patients has improved significantly in recent years, mortality rates for these patients remain several times higher than those for individuals in the general population [1]. Comorbidities in dialysis patients, which are highly prevalent and characterized by simultaneous coexistence, are often associated with substantially high mortality rates and socio-economic burden [1–6]. Thus, the prognostic impacts of comorbidities may influence diverse decisions during clinical practice and should be adjusted as strong confounding factors in statistical analyses. Consequently, comorbidities should be carefully assessed and summarized by an index that is intuitive to physicians for clinical and research uses. More importantly, such an index should reflect current advances regarding the management and outcomes of comorbidities and should be updated accordingly.

Among the current indices, the Charlson comorbidity index (CCI) which was developed in 1987, is the most popular and simple. It has been successfully validated in diverse chronic disease populations, including dialysis patients [7–10]. Nevertheless, there are several reasons to recalibrate and subsequently validate the index in dialysis patients to obtain better predictions regarding their mortality. Because the assigned weights of comorbidities in the CCI were generated from a general medical inpatient population, some comorbid conditions might have different significance in predicting mortality among dialysis patients [11, 12]. In addition, the management of dialysis patients and their comorbidities has advanced considerably, and the contributions of comorbidities to mortality rates might have changed as well [13]. Although some previous studies proposed modification of the CCI in dialysis patients, these modified indices were developed based on relatively small numbers of dialysis patients and were not externally validated through appropriate performance analyses [14, 15].

In the present study, we aimed to modify the CCI by recalibrating the weights of comorbidities in the CCI using a Korean national population-based registry database, to validate the index in a separate nationwide prospective end-stage renal disease (ESRD) cohort, and to compare its performance with the CCI.

## Materials and Methods

### Data source

#### Development cohort

Deidentified data on all Koreans aged ≥18 years who received hemodialysis (HD) as their first modality of renal replacement therapy between January 2005 and December 2008 were obtained from the Korean Health Insurance database (n = 28,032). Data on all Korean HD patients could be reviewed because Koreans are obligated to register with the national insurance system, which consists of health insurance and medical aid. Subjects who died (n = 2,751), changed their modality of renal replacement (n = 229), or underwent kidney transplantation (n = 314) within 3 months after starting HD were excluded. Therefore, a total of 24,738 patients were included in the analysis to develop a modified CCI for incident HD patients (mCCI-IHD).

#### Validation cohort

All patients aged ≥18 years who started HD as their first modality of renal replacement therapy between August 2008 and December 2012 in 31 centers of the Clinical Research Center (CRC) for ESRD and remained on HD for more than 3 months were initially screened for the validation cohort (n = 1,165). The CRC for ESRD is a nationwide multicenter prospective cohort of patients with ESRD in Korea (NCT00931970). Patients who died within 3 months of the commencement of hemodialysis (n = 65) were excluded from the analyses. A total of 1,100 incident HD patients were included to externally validate the mCCI-IHD.

### Data collection

Comorbidities were defined based on the International Classification of Diseases, 10^th^ Revision (ICD-10) (S1 Table) [16]. For the development study, information on comorbidities that constitute the CCI, including myocardial infarction (MI), congestive heart failure (CHF), peripheral vascular disease (PVD), cerebrovascular disease, dementia, chronic pulmonary disease, connective tissue disease, ulcer disease, mild liver disease, diabetes, hemiplegia, diabetes with end-organ damage, any tumor (including leukemia and lymphoma), moderate to severe liver disease and metastatic solid tumors were derived by extracting ICD-10 codes leading up to the initiation of dialysis therapy [17]. The comorbidity data of the validation cohort were ascertained by clinicians of CRC for ESRD through history taking and review of electronic health record. Acquired immunodeficiency syndrome was not included because no patient was so diagnosed in either cohort. Mortality data were obtained from the Statistics Korea and National Health Insurance Claims databases. In the validation cohort, demographic characteristics included body mass index (BMI), health security system, primary cause of ESRD, and timing of referral to nephrology; early referral was defined by HD initiation occurring more than 12 months after the first encounter with a nephrologist. The laboratory data included pre-dialysis fasting blood samples taken 1 month after the initial HD and included the following: hemoglobin, albumin, calcium, and phosphorus.

### Statistical analyses

All statistical analyses were performed using SAS version 9.3 (SAS Institute, Cary, NC) or R version 3.0.3 (R Development Core Team, Austria). The data were summarized according to means±SDs or medians±IQRs for continuous variables and proportions for categorical variables. The multivariate Cox proportional hazards model was used to develop new comorbidity weights. We computed adjusted HRs and *β* coefficients while accounting for age, sex, and all 15 comorbidities simultaneously. We excluded an unnecessary item of severe renal disease given its presence in all of the study subjects. Prognostic weights were derived by dividing each *β* coefficient by the lowest one with statistical significance and rounding to the nearest integer [18]. Following this procedure, a comorbidity score was calculated for each subject by summing up the weights. The prognostic powers of the CCI and mCCI-IHD were compared by estimating Kaplan-Meier curves and multivariate Cox regression models. To compare the capacity for discrimination between the indices, a ***c*** statistic was calculated using a receiver-operating characteristic curve [19]. Additionally, the continuous net reclassification improvement (cNRI) was calculated to determine the extent to which the mCCI-IHD improved mortality predictions compared to the CCI [20], age-adjusted CCI [21] and previously modified CCI (mCCI) in ESRD patients [15]. Logistic regression models were first adopted to calculate cNRI. In the fitted logistic models, the same covariates were entered by adding two scores separately using the function *improveprob* in R [22]. In all analyses, a *P*<0.05 was considered statistically significant.

### Ethical aspects

The study protocol complied with the Declaration of Helsinki and received full approval from the institutional review board (IRB) at Seoul National University Hospital (H-1405-060-579). The study protocol of CRC for ESRD was approved by the IRB at each participating center [The Catholic University of Korea, Bucheon St. Mary’s Hospital; The Catholic University of Korea, Incheon St. Mary’s Hospital; The Catholic University of Korea, Seoul St. Mary’s Hospital; The Catholic University of Korea, St. Mary’s Hospital; The Catholic University of Korea, St. Vincent’s Hospital; The Catholic University of Korea, Uijeongbu St. Mary’s Hospital; Cheju Halla General Hospital; Chonbuk National University Hospital; Chonnam National University Hospital; Chung-Ang University Medical Center; Chungbuk National University Hospital; Chungnam National University Hospital; Dong-A University Medical Center; Ehwa Womens University Medical Center; Fatima Hospital, Daegu; Gachon University Gil Medical Center; Inje University Pusan Paik Hospital; Kyungpook National University Hospital; Kwandong University College of Medicine, Myongji Hospital; National Health Insurance Corporation Ilsan Hospital; National Medical Center; Pusan National University Hospital; Samsung Medical Center, Seoul; Seoul Metropolitan Government, Seoul National University, Boramae Medical Center; Seoul National University Hospital; Seoul National University, Bundang Hospital; Yeungnam University Medical Center; Yonsei University, Severance Hospital; Yonsei University, Gangnam Severance Hospital; Ulsan University Hospital; Wonju Christian Hospital (in alphabetical order)], and all patients provided their written informed consent.

## Results

### Development of the new comorbidity index (mCCI-IHD) for the prediction of mortality

Baseline characteristics of the development cohort are listed in Table 1. The mean age at initiation of HD therapy was 57.9 years and 59.5% of patients were men. The median follow-up duration was 47.7 months. A total of 76.3% of the subjects had one or more comorbidities. Among the 15 comorbidities, diabetes (with and without end-organ damage) was most prevalent (49.6%), followed by chronic pulmonary disease (16.3%), CHF (14.1%), and cerebrovascular disease (13.0%). The survival rates of the subjects were 92.4%, 87.5%, 78.9%, and 72.3% at 6 months and 1, 2, and 3 years of follow-up, respectively.

**Table 1:** Baseline characteristics of the development and validation cohorts.

Variables	Development cohort	Validation cohort
	(n = 24,738)	(n = 1,100)
Age (years, mean (SD))	57.9 (14.2)	60.7 (14.3)
Age (N (%))		
<50 years	6,831 (27.7)	227 (20.6)
50–59 years	5,641 (22.9)	247 (22.5)
60–69 years	6,607 (26.8)	290 (26.4)
≥70 years	5,659 (22.10)	336 (30.6)
Male sex (N (%))	14,708 (59.6)	663 (60.3)
Number of death (N (%))	9,431 (38.2)	143 (13.0)
Follow-up duration (months, median (IQR))	47.7 (32.10)	22.0 (20.1)
Health security system (N (%))		
National health insurance	21,277 (86.0)	862 (78.4)
Medical aid^a^	3,461 (14.0)	212 (19.3)
Others	0	26 (2.4)
Comorbidity (N (%))		
No comorbidity	5,866 (23.7)	236 (21.5)
Ulcer disease	3,682 (14.9)	73 (6.6)
Chronic pulmonary disease	4,027 (16.3)	92 (8.4)
Peripheral vascular disease	1,491 (6.0)	114 (10.4)
Mild liver disease	2,508 (10.1)	61 (5.6)
Myocardial infarct	797 (3.2)	68 (6.2)
Connective tissue disease	688 (2.8)	93 (8.5)
Congestive heart failure	3,500 (14.2)	149 (13.6)
Diabetes	1,712 (6.9)	164 (14.9)
Hemiplegia	395 (1.6)	16 (1.5)
Cerebrovascular disease	3,209 (13.0)	144 (13.1)
Diabetes with end-organ damage	10,556 (42.7)	466 (42.4)
Dementia	377 (1.5)	4 (0.4)
Any tumor (including leukemia and lymphoma)	1,400 (5.7)	77 (7.0)
Moderate to severe liver disease	206 (0.8)	40 (3.6)
Metastatic solid tumor	253 (1.0)	4 (0.4)
CCI score (median (IQR))	4 (1)	4 (3)
Referral to nephrologists (N (%))	-	
Early referral	-	628 (57.1)
Late referral	-	405 (36.8)
Missing	-	67 (6.1)
Primary cause of end-stage renal disease (N (%))	-	
Diabetes	-	570 (51.8)
Chronic glomerulonephritis	-	142 (12.9)
Hypertension	-	170 (15.5)
Hereditary, congenital disease, and others	-	59 (5.4)
Unknown	-	159 (14.5)
Body mass index (kg/m^2^, mean (SD))	-	23.20 (3.5)
Hemoglobin (g/dL, mean (SD))	-	8.8 (1.0)
Albumin (g/dL, mean (SD))	-	3.33 (0.6)
Calcium (mg/dL, mean (SD))	-	7.8 (0.6)
Phosphorus (mg/dL, mean (SD))	-	5.3 (1.2)

CCI, Charlson comorbidity index.

^a^program for low income earners who received livelihood assistance

Table 2 lists the adjusted HRs, *β* coefficients and weights for each comorbidity. All comorbidities except ulcers significantly predicted mortality. The relative weights of comorbidities were calculated by their ratios with the lowest *β* coefficient of chronic pulmonary disease. As expected, metastatic solid tumors were the strongest predictor of mortality among comorbidities; accordingly, the weight assigned to this condition was the highest. Moderate to severe liver disease, any tumor (including leukemia and lymphoma), diabetes with end-organ damage, dementia, and cerebrovascular disease followed consecutively.

**Table 2:** Weights for comorbidities in the development cohort.

	Development cohort (n = 24,738)			
	HR (95% CI)^a^	*P* value	*β* Coefficient	Weight
Age				
<50 years	Ref			
50–59 years	1.84 (1.71–1.99)	<0.0001		
60–69 years	3.08 (2.86–3.31)	<0.0001		
≥70 years	5.40 (5.03–5.80)	<0.0001		
Sex (female vs. male)	0.88 (0.85–0.92)	<0.0001		
Comorbidity				
Ulcer disease	0.96 (0.91–1.02)	0.2044	-0.041	0
Chronic pulmonary disease	1.10 (1.04–1.16)	0.0004	0.095	1
Peripheral vascular disease	1.10 (1.02–1.19)	0.0117	0.095	1
Mild liver disease	1.19 (1.11–1.27)	<0.0001	0.174	2
Myocardial infarct	1.19 (1.05–1.34)	0.0045	0.174	2
Connective tissue disease	1.21 (1.10–1.33)	0.0001	0.191	2
Congestive heart failure	1.24 (1.18–1.31)	<0.0001	0.215	2
Diabetes	1.27 (1.18–1.37)	<0.0001	0.239	3
Hemiplegia	1.29 (1.12–1.47)	0.0002	0.255	3
Cerebrovascular disease	1.34 (1.27–1.41)	<0.0001	0.293	3
Diabetes with end-organ damage	1.38 (1.22–1.56)	<0.0001	0.322	3
Dementia	1.40 (1.34–1.46)	<0.0001	0.336	4
Any tumor (including leukemia and lymphoma)	1.56 (1.45–1.68)	<0.0001	0.445	5
Moderate to severe liver disease	2.46 (2.05–2.95)	<0.0001	0.900	9
Metastatic solid tumor	3.55 (3.08–4.10)	<0.0001	1.267	13

^a^Adjusted for age, sex and all comorbidities

**Fig 1:**
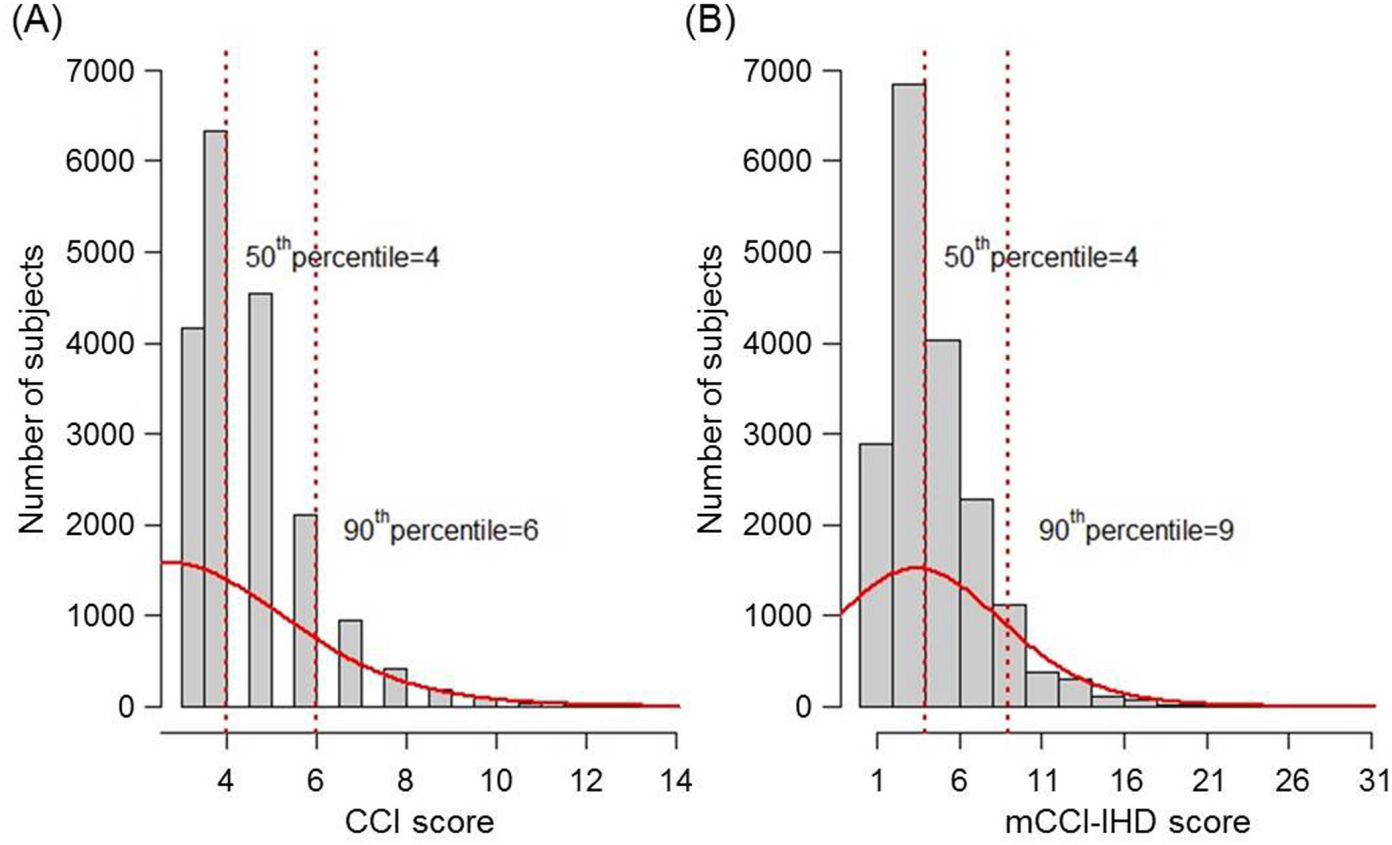
Distribution of the CCI and mCCI-IHD scores for the development cohort. (A) Distribution of the CCI scores (n = 18,872), excluding patients with no comorbidity (n = 5,866). (B) Distribution of the mCCI-IHD scores (n = 18,087), excluding patients with no comorbidity (n = 6,651). The y-axis shows the number of subjects. The solid line represents a density curve, calculated by approximation, to identify the overall pattern and deviation. The vertical dotted lines (red) represents the 50^th^ and 90^th^ percentile values. CCI, Charlson comorbidity index; mCCI-IHD, modified Charlson comorbidity index for incident hemodialysis patients.

**Fig 2:**
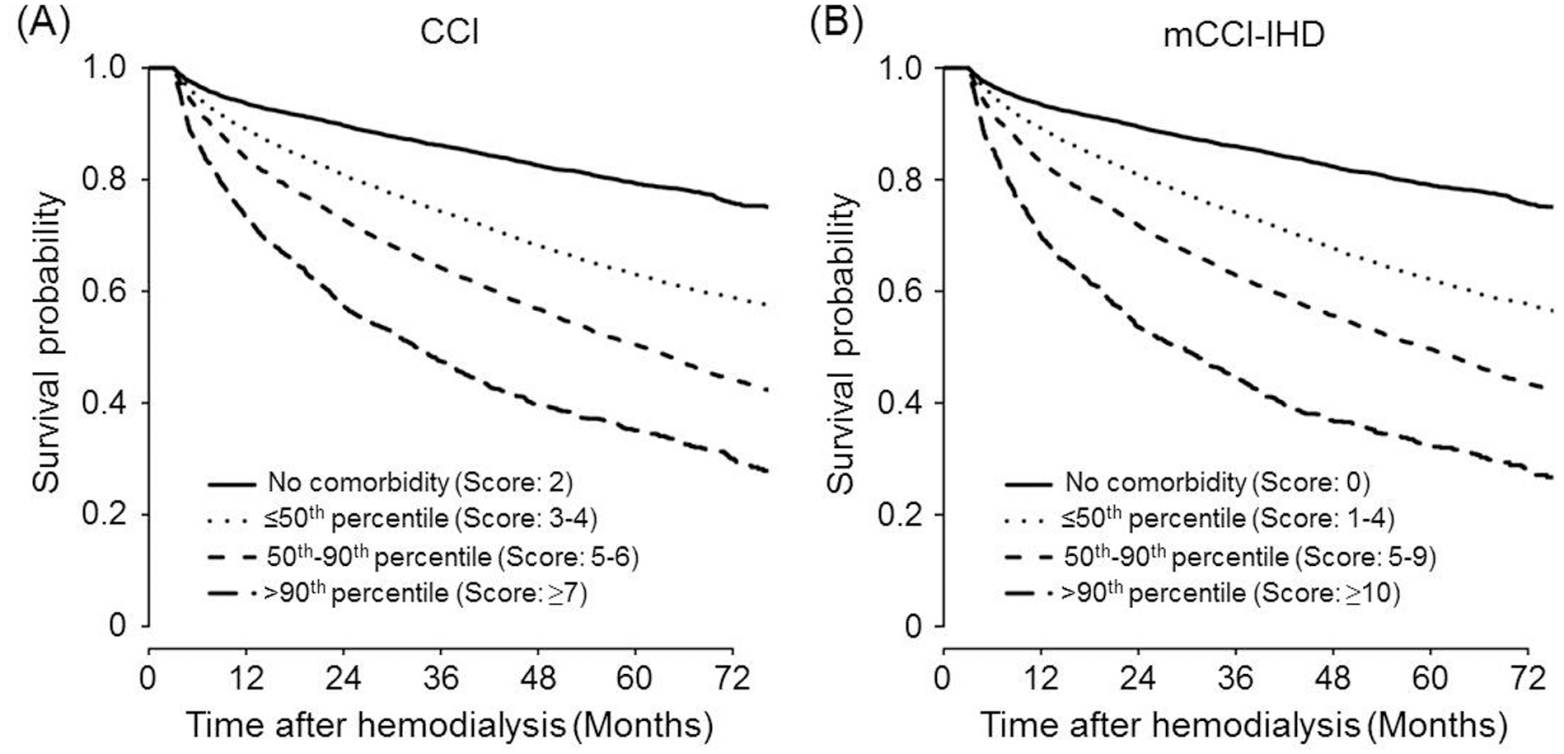
Survival curves obtained using the Kaplan-Meier method in the development cohort differentiated by 4 risk groups for CCI (A) and mCCI-IHD (B). CCI, no comorbidity (n = 5,866, score 2); ≤50^th^ percentile (n = 10,501, scores 3–4); 50^th^-90^th^ percentile (n = 6,663, scores 5–6); and >90^th^ percentile (n = 1,708, score ≥7). mCCI-IHD, no comorbidity (n = 6,651, score 0); ≤50^th^ percentile (n = 9,740, scores 1–4); 50^th^-90^th^ percentile (n = 7,072, scores 5–9); and >90^th^ percentile (n = 1,275, score ≥10). CCI, Charlson comorbidity index; mCCI-IHD, modified Charlson comorbidity index for incident hemodialysis patients.

The summed scores of the original and recalibrated weights were applied to each patient in the development cohort. In the Cox proportional hazards model adjusted for age and sex, the HRs of the CCI and mCCI-IHD scores for mortality were 1.42 (95% CI, 1.39–1.45; *P*<0.0001) and 1.72 (95% CI, 1.66–1.78; *P*<0.0001), respectively. Based on the CCI and mCCI-IHD scores, the subjects were further collapsed into 4 risk groups according to the following criteria: no comorbidity, ≤50^th^ percentile, 50^th^-90^th^ percentile, and >90^th^ percentile. To determine the cutoff values of the comorbidity scores for each risk group, we examined the distributions of the scores using a histogram (Fig 1). The cutoff values of the CCI scores corresponding to the 50^th^ and 90^th^ percentiles were 4 and 6, whereas those of the mCCI-IHD scores corresponding to the same percentiles were 4 and 9. Fig 2 shows the Kaplan-Meier curves for the CCI and mCCI-IHD differentiated by the 4 risk groups in the development cohort. The appearance of survival curves across increasing comorbidity scores was similar for both indices, with anticipated poorer survival for higher scores (*P*<0.001).

### Application and validation of the mCCI-IHD

**Fig 3:**
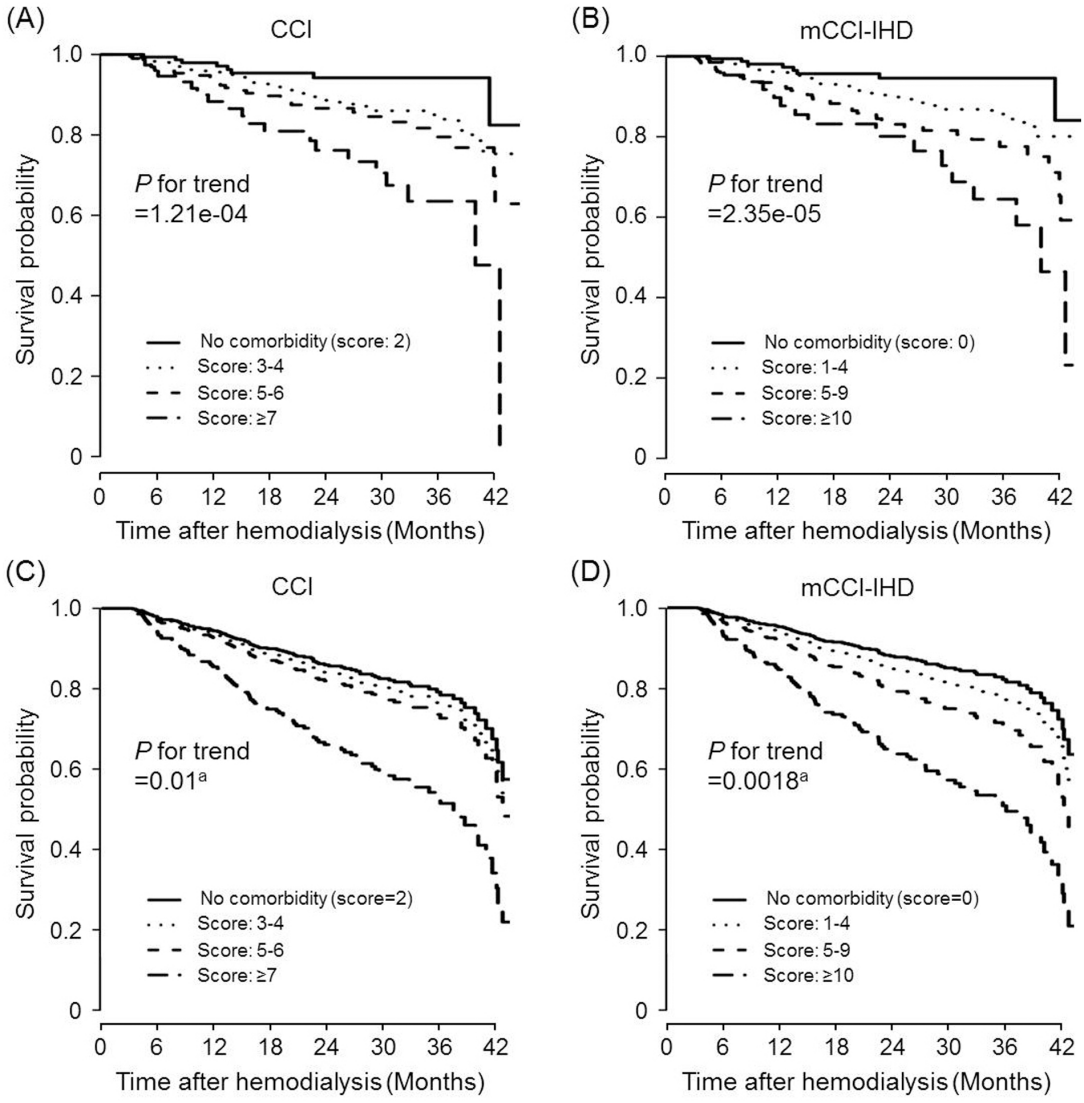
Survival curves obtained using the Kaplan-Meier method (A and B) and Cox regression analysis (C and D) in the validation cohort differentiated by the 4 risk groups of CCI (A and C) and mCCI-IHD (B and D). CCI, score 2 (n = 236, 21.5%); scores 3–4 (n = 537, 48.8%); scores 5–6 (n = 234, 21.3%); and score ≥7 (n = 93, 8.5%). mCCI-IHD, score 0 (n = 247, 22.5%); scores 1–4 (n = 480, 43.6%); scores 5–9 (n = 290, 26.4%); and score ≥10 (n = 83, 7.5%). ^a^Adjusted for age, sex, health security system, timing of referral to nephrologist, primary cause of end-stage renal disease, body mass index, hemoglobin, albumin, calcium, and phosphorus. CCI, Charlson comorbidity index; mCCI-IHD, modified Charlson comorbidity index for incident hemodialysis patients.

Table 1 lists the characteristic of the validation cohort (n = 1,100). The majority of subjects were men, with a mean age of 60.7 years. A total of 143 mortalities (13.0%) were observed during the median follow-up of 22.0 months. Along with the development cohort, diabetes was the most common comorbidity (57.3%), followed by CHF (13.6%), cerebrovascular disease (13.1%), and PVD (10.4%). The mCCI-IHD and CCI scores were then calculated for patients in the validation cohort. Fig 3 shows the survival curves obtained using the Kaplan-Meier method and multivariate Cox regression analysis (adjusted for age, sex, health security system, timing of referral to nephrologists, primary cause of ESRD, BMI, hemoglobin, albumin, calcium, and phosphorus) in the validation cohort, which were differentiated by the 4 risk groups of the CCI and mCCI-IHD. The risk groups based on each index provided some discrimination for survival, indicating that increasing comorbidity index scores were associated with lower cumulative survival. However, multivariate-adjusted survival curves for the mCCI-IHD risk groups showed a highly significant linear gradient and were quite well separated, whereas the model using the CCI seemed less able to distinguish among the three lowest-risk groups (Fig 3).

As detailed in Table 3, increased ***c*** statistics and cNRI for the mCCI-IHD supported better performance characteristics of the mCCI-IHD compared to the CCI. The mCCI-IHD exhibited modest but significant increases in ***c*** statistics compared with the CCI at 6 months and 1 year but not at 2 years in multivariate analyses adjusted for age, sex, health security system, timing of referral to nephrologist, primary cause of ESRD, BMI, hemoglobin, albumin, calcium, and phosphorus. The analyses using cNRI revealed that the mCCI-IHD improved net mortality risk reclassification by 24.6% (95% CI, 2.5–46.7; *P* = 0.03), 26.2% (95% CI, 1.0–51.4; *P* = 0.04) and 42.8% (95% CI, 4.9–80.8; *P* = 0.03) relative to the CCI at 6 months and 1 and 2 years, respectively, after adjustment for the above-mentioned confounders. This finding indicates that the mCCI-IHD may provide better risk stratification for mortality in incident HD patients than the CCI. Additionally, the mCCI-IHD also improved the predictability for mortality compared with the age-adjusted CCI (cNRI, 25.6%; *P* = 0.04 and cNRI, 45.7%; *P* = 0.02 at 1 and 2 years, respectively) and the mCCI (cNRI, 48.0%; *P* = 0.02 at 2 years) after adjusting the confounding factors.

**Table 3:** Model performance of the mCCI-IHD at various time points during the follow-up of the validation cohort.

			*c* Statistic^a^ (95% CI)	*P* value	cNRI^a^ (%, 95% CI)	*P* value
CCI^[^ ^20^ ^]^ versus mCCI-IHD	6-month follow-up (n = 798)	CCI	0.61 (0.55–0.66)			
	6-month follow-up (n = 798)	mCCI-IHD	0.64 (0.58–0.69)	0.04	24.6 (2.5–46.7)	0.03
	1-year follow-up (n = 638)	CCI	0.61 (0.55–0.68)			
	1-year follow-up (n = 638)	mCCI-IHD	0.65 (0.58–0.71)	0.04	26.2 (1.0–51.4)	0.04
	2-year follow-up (n = 399)	CCI	0.66 (0.56–0.76)			
	2-year follow-up (n = 399)	mCCI-IHD	0.69 (0.59–0.79)	0.29	42.8 (4.9–80.8)	0.03
age-adjusted CCI^[^ ^21^ ^]^ versus mCCI-IHD	6-month follow-up (n = 798)	Age-adjusted CCI	0.66 (0.59–0.70)			
	6-month follow-up (n = 798)	mCCI-IHD	0.64 (0.58–0.68)	0.69	16.7 (-5.7–39.0)	0.14
	1-year follow-up (n = 638)	Age-adjusted CCI	0.65 (0.58–0.71)			
	1-year follow-up (n = 638)	mCCI-IHD	0.65 (0.58–0.71)	0.95	25.6 (0.25–51.0)	0.04
	2-year follow-up (n = 399)	Age-adjusted CCI	0.67 (0.56–0.77)			
	2-year follow-up (n = 399)	mCCI-IHD	0.69 (0.59–0.79)	0.43	45.7 (8.8–82.6)	0.02
mCCI^[^ ^15^ ^]^ versus mCCI-IHD	6-month follow-up (n = 798)	mCCI	0.64 (0.58–0.70)			
	6-month follow-up (n = 798)	mCCI-IHD	0.64 (0.58–0.69)	0.69	6.4 (-15.6–28.4)	0.57
	1-year follow-up (n = 638)	mCCI	0.64 (0.57–0.71)			
	1-year follow-up (n = 638)	mCCI-IHD	0.65 (0.58–0.71)	0.82	7.2 (-17.4–31.9)	0.57
	2-year follow-up (n = 399)	mCCI	0.67 (0.58–0.77)			
	2-year follow-up (n = 399)	mCCI-IHD	0.69 (0.59–0.79)	0.50	48.0 (9.3–86.7)	0.02

cNRI, continuous net reclassification improvement; CCI, Charlson comorbidity index; mCCI-IHD, modified Charlson comorbidity index for incident hemodialysis patients.

^a^Adjusted for age, sex, health security system, timing of referral to nephrologist, primary cause of end-stage renal disease, body mass index, hemoglobin, albumin, calcium, and phosphorus.

## Discussion

In the present study, we modified the CCI using population-based registry data that included nearly all Korean patients who began HD between 2005 and 2008 to develop a comorbidity index that provides better risk stratification in incident HD patients. In addition, we validated the usefulness of the modified index in an independent prospective cohort by comparing its performance with the original index. To our knowledge, this is the first study to recalibrate the CCI using a large sample of incident HD patients and to validate the index using an independent nationwide prospective observational cohort of ESRD patients.

Despite highly prevalent comorbidities and their influence on mortality rates in ESRD patients, it has not yet been determined which comorbidities significantly affect mortality and what weight each comorbidity carries when predicting mortality. Therefore, many previous studies have examined the influence of comorbidity on survival in patients with ESRD [23–25], and several comorbidity instruments such as the CCI [14, 26], the index of co-existent disease (ICED) [27], the Davies index [14], and the Khan index [4, 14] have been validated in dialysis patients. Among the indices, the ICED has been shown to be a valid method of assessing comorbidity in dialysis patients and appears superior to the others based on its discriminatory ability [23, 28, 29]. However, this index consists of more than 100 variables, which makes it largely inapplicable for clinical practice or statistical analysis. The Davies index, which consists of 7 comorbidities, is a simple and clinically applicable instrument [30]. However, all the comorbidities in this index are assigned equal weights based on ill-defined criteria, which means there might be a lack of consideration for the differential impacts of the comorbidities on mortality. The Khan index also has limitations due to its reliance on weak evidence for selecting comorbidities as predictors of mortality and its use of equal weights for each comorbidity [4]. In contrast, the CCI is the most widely used and validated because of its convenience and relatively well-defined weights for comorbidities. Nevertheless, the CCI remains highly disputed as an index to be applied directly to current ESRD populations because the weights for certain comorbidities in the CCI may be overestimated when considering the contemporary management of comorbidities. Thus, the applicability of the CCI to current clinical practice or statistical analysis may be tenuous. Indeed, the adjusted HRs of comorbidities in the present study, especially in the case of cardiovascular comorbidities, which are the main causes of death in this population, were lower than those in the original study of Charlson *et al*. [26] (i.e., MI [1.21 versus 1.40], CHF [1.24 versus 1.30], PVD [1.10 versus 1.30], hemiplegia [1.29 versus 1.30], and cerebrovascular disease [1.34 versus 1.40]). Similar patterns were found when the results presented here were compared with HRs in a study from the early 2000s, which modified the CCI based on a relatively small number of ESRD patients (n = 237) [15]. Conversely, a recent study using data from the Dialysis Outcomes and Practice Patterns Study revealed HRs for cardiovascular comorbidities similar to those calculated in the present study, suggesting advances in the management of the comorbidities and subsequent improved outcomes since the 1980s [3]. While the HRs have decreased, the weights of the cardiovascular comorbidities were assigned higher values in the mCCI-IHD than in the CCI (i.e., MI [2 versus 1], CHF [2 versus 1], PVD [1 in both indices], hemiplegia [3 versus 2], and cerebrovascular disease [3 versus 1]). Given that cardiovascular comorbidities are the most common cause of death in this population [1, 31], this finding suggests that the mCCI-IHD properly reflects the differential significance of cardiovascular comorbidities in this population, thereby providing superior performance in mortality risk stratification.

The results of performance analyses demonstrated that the mCCI-IHD exhibited significant but modest improvements in ***c*** statistics at 6 months and 1 year compared with the CCI, exhibiting increases of only 0.03–0.04. In this regard, it has become increasingly recognized that increases in ***c*** statistics of a test model with a large effect size may be small when compared to a strong baseline model such as the CCI [32, 33]. Thus, even though it is a popular metric through which to capture discrimination, ***c*** statistics may not be useful for comparing the performance of two risk prediction models [34–36]. In contrast, cNRI has been proposed to assess the degree of improvement in discrimination of risk prediction between the models and is not strongly affected by the effect size of the baseline model [32, 33]. Furthermore, this metric can capture the degree of improvement in discrimination after accounting for correlation with other variables in the risk prediction model [32]. Therefore, we additionally calculated the cNRI to quantify the magnitude of correct reclassification, and the mCCI-IHD showed significant positive values compared with the CCI, age-adjusted CCI, and mCCI after adjustment for socio-economic, clinical and laboratory parameters related to the survival of HD patients [1, 37–40]. These findings indicate that the mCCI-IHD may provide better risk stratification for mortality in incident HD patients compared with the original and previously modified CCI.

Our study had limitations. First, our results from the development cohort were derived from the administrative database of the national insurance system. Although the administrative data have advantages, such as conservation of time and resources and consistency in diagnosis due to the uniform use of the ICD-10, there may be inappropriate diagnoses due to the drawbacks of the coding system and the physicians’ preference toward diagnostic codes with higher reimbursements [41]. In addition, this dataset did not contain parameters such as biochemical measures and socioeconomic status. Therefore, we could not adjust these variables in the analysis for recalibrating the weights of comorbidities. Second, because there was no patient with acquired immunodeficiency syndrome in either cohort, this condition was not taken into consideration. Third, we excluded those subjects who died within 3 months after starting HD to rule out patients with critical illness or terminal conditions with renal failure. However, this could bias the assigned severity weights of the comorbidities because of a higher mortality in early dialysis period. Fourth, the mCCI-IHD was derived from a single ethnic group of Korean HD patients. Therefore, generalizability of the index can be limited. Nonetheless, a major strength of the present study is that patient mortality was determined using a large nationwide, population-based data set. Furthermore, the modified index was validated using an independent database. However, additional validation using disease-specific cohorts and other national data may be necessary.

In conclusion, we propose a new scoring system that provides better risk stratification for mortality in incident HD patients compared with the CCI after adjusting for socio-economic, clinical and laboratory parameters. Therefore, it may be a preferred index for use in clinical practice and the statistical analysis of epidemiological studies.

## Supporting Information

S1 Table ICD-10 coding algorithms for Charlson comorbidities. (DOCX)
